# A Sensor Fusion Method for Tracking Vertical Velocity and Height Based on Inertial and Barometric Altimeter Measurements

**DOI:** 10.3390/s140813324

**Published:** 2014-07-24

**Authors:** Angelo Maria Sabatini, Vincenzo Genovese

**Affiliations:** The BioRobotics Institute, Scuola Superiore Sant'Anna, Viale Rinaldo Piaggio, Pontedera 34 56025, Pisa, Italy; E-Mail: vincenzo.genovese@sssup.it

**Keywords:** sensor fusion, inertial sensors, barometric altimeters, motion tracking, Kalman filtering

## Abstract

A sensor fusion method was developed for vertical channel stabilization by fusing inertial measurements from an Inertial Measurement Unit (IMU) and pressure altitude measurements from a barometric altimeter integrated in the same device (baro-IMU). An Extended Kalman Filter (EKF) estimated the quaternion from the sensor frame to the navigation frame; the sensed specific force was rotated into the navigation frame and compensated for gravity, yielding the vertical linear acceleration; finally, a complementary filter driven by the vertical linear acceleration and the measured pressure altitude produced estimates of height and vertical velocity. A method was also developed to condition the measured pressure altitude using a whitening filter, which helped to remove the short-term correlation due to environment-dependent pressure changes from raw pressure altitude. The sensor fusion method was implemented to work on-line using data from a wireless baro-IMU and tested for the capability of tracking low-frequency small-amplitude vertical human-like motions that can be critical for stand-alone inertial sensor measurements. Validation tests were performed in different experimental conditions, namely no motion, free-fall motion, forced circular motion and squatting. Accurate on-line tracking of height and vertical velocity was achieved, giving confidence to the use of the sensor fusion method for tracking typical vertical human motions: velocity Root Mean Square Error (RMSE) was in the range 0.04–0.24 m/s; height RMSE was in the range 5–68 cm, with statistically significant performance gains when the whitening filter was used by the sensor fusion method to track relatively high-frequency vertical motions.

## 1. Introduction

Sensor fusion methods combine data from disparate sources of information in a way that should give better performance than can be achieved when each source of information is used alone. The design of systems based on sensor fusion methods requires the availability of complementary sensors in order that the disadvantages of each sensor are overcome by the advantages of the others. An interesting application niche for sensor fusion methods—the one investigated in this paper—is motion tracking. Several sensor technologies are available for motion tracking, however none of them, taken alone, can be considered the absolute winner, especially when motion is to be tracked without restrictions in space and time [1], and cost and compliance issues tend to restrict the range of potential candidates for applications like human motion tracking in biomedicine and healthcare.

Micro Electro-Mechanical System (MEMS) sensors are well suited for motion tracking in human-centric applications, such as pedestrian navigation and ambulatory human motion monitoring, in several regards (*i.e.*, cost, form factor, power consumption) [2]. However their output signals are prone to noise and drift to the extent that good tracking of either orientation or position are difficult to achieve [3]: for instance, a tracking system relying on stand-alone measurements from MEMS inertial sensors suffers from integration drifts that may lead to positioning errors of 2 m after only 10 s of operation. It is customary to stabilize the position and orientation of MEMS inertial sensors using sensor fusion methods: GPS [4], ultra-wideband (UWB) radio [5], vision [6] are popular sensors that have been widely investigated for being integrated in inertial navigation systems. Barometric altimeters have also been proposed [5,7,8], in particular for stabilizing the position in the vertical direction (height tracking). Henceforth, the integration of a barometric altimeter with an Inertial Measurement Unit (IMU) embedding a tri-axial gyroscope and a tri-axial accelerometer is referred to as a baro-IMU. Good portability and ready market availability of low-cost sensor devices with proprietary sensor fusion software are appealing advantages of baro-IMU technology for several industrial, defense and research applications. Prospective applications can also be foreseen for human motion tracking, however custom sensor fusion software still needs to be developed.

Barometric altimeters allow one to estimate the height of an object above a given reference level, e.g., the sea level from air pressure measurements—A popularly used term for this height is pressure altitude. In the context of human-centric applications, barometric altimeters found use within multi-sensor pedestrian navigation systems and activity monitors, for improving performance of dead-reckoning algorithms or classifiers of human motion patterns: e.g., they were used to detect the moving styles of going up/down the stairs or in an elevator [9]; to determine the correct floor of a user in a multi-storey building [10]; to detect stair ascent-descent in ambulatory monitors designed for estimating the energy expenditure incurred during activities of daily living, including stair-walking [11,12]. More recently, it was proposed that the measured pressure altitude might help improving accelerometry-based fall detection systems [13–15]. However, rapid changes in pressure that are uncorrelated with altitude can be generated, outdoors, by unpredictable atmospheric conditions, and, indoors, by opening/closing of windows or doors and by air conditioning systems [16]. The consequence is that barometric altimeters tend to be very noisy, and their accuracy is rather poor; when absolute altitude has to be estimated, the use of a reference barometric altimeter at a known and constant altitude has been proposed in the effort to obtain accurate measurements from a moving barometric altimeter (differential barometry) [16]. Oftentimes, a single barometric altimeter can be used for tracking relative changes in height [8], to which we adhere in this paper. Barometric pressure technology allowing for altitude changes such as those occurring, e.g., in transitions between sitting and standing or during falls to be measured with sufficient accuracy may thus represent a valuable add-on to inertial sensor-based activity monitors [17].

Two main approaches are pursued in the development of sensor fusion methods for motion tracking, namely tightly coupled and loosely coupled. The tightly coupled systems combine all sensor data into a single filter [5], while the loosely coupled systems revolve around different filtering modules for their operation [8]. In this paper we opted for the development of a loosely coupled sensor fusion method revolving around a two-stage cascade [8].

First, a filtering module consisting of a quaternion-based Extended Kalman Filter (EKF) estimated the attitude of the body frame using inertial sensors [18]; the estimated inclination allowed to rotate the specific force measured by the tri-axial accelerometer from the body frame to the navigation frame, in preparation for gravity compensation. Second, the estimated vertical linear acceleration and the output provided by the barometric altimeter fed another filtering module—a complementary filter that estimated the vertical velocity and height in the navigation frame [19].

Different means of pre-processing the measured pressure altitude were also implemented and tested, namely *M*-point moving average filters cascaded or not with a whitening filter, whose design was inspired by our studies on stochastic approaches to the problem of modeling the noise in barometric altimeters [20]. The whitening filter attempted to remove the serial correlation of the noise due to environment-dependent short-term pressure changes that may affect short-time tracking accuracy, *i.e.*, when the motions to be tracked last up to few minutes. One goal of this paper was to demonstrate the extent to which different methods of preprocessing noisy pressure altitude measurements can help to stabilize the integration of specific force measurements, leading to accurate estimates of vertical velocity and possibly height under conditions typical of human motions.

The sensor fusion methods were applied to data from a wireless baro-IMU of our own design. Several tests were performed in different experimental settings, namely no-motion, free-fall motion, forced circular motion and squatting. When possible, reference data were generated and used for performance assessment. Accurate tracking of height and vertical velocity was demonstrated: velocity Root Mean Square Error (RMSE) was in the range 0.04–0.24 m/s; height RMSE was in the range 5–68 cm, with statistically significant performance gains when the whitening filter was used to track relatively high-frequency vertical motions.

## 2. Experimental Section

### 2.1. Algorithm Development

**Figure 1.**
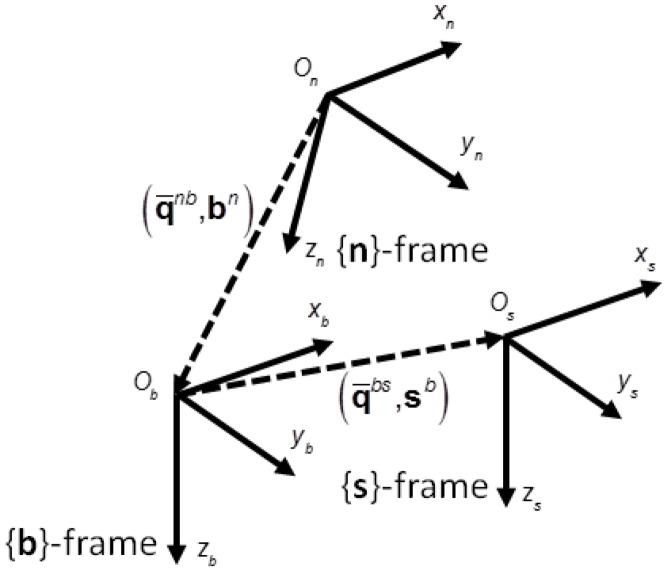
The reference frames involved in the experimental set-up.

Three reference frames were used in the experimental setup of this paper (Figure 1):

- Navigation frame {**n**}—this was a North East Down (NED) frame, in which the Earth's gravity was assumed known. The goal of the developed algorithm was to estimate the vertical velocity and height of the baro-IMU case in {**n**}. Vertical meant locally aligned with the direction of the gravity acceleration at the origin of {**n**}.
- Sensor frame {**s**}—this was the reference frame in which the inertial sensors provided their measurements. The axes of the sensor frame were aligned with the sensitive axes of the tri-axial gyro and the tri-axial accelerometer.
- Body frame {**b**}—this was the reference frame of the rigid body to which the baro-IMU case was fastened.{**b**}was related to{**s**}through a rigid transformation (rotation matrix and translation).

The following notation was used to express the relation between two frames, for instance {**n**} and {**b**}: **C***^nb^* and ${\bar{\text{q}}}^{nb}={\left[{\left(q^{nb}\right)}^{T}q_{4}^{nb}\right]}^{T}$ denoted, respectively, the rotation matrix and the quaternion from {**b**} to {**n**} (**q***^nb^* was the vector part and $q_{4}^{nb}$ was the scalar part of **q̄**^*nb*^), **b***^n^* was the position of the origin of {**b**} *O_b_* relative to the origin of {**n**} *O_n_*, resolved in {**n**}. We assumed that, at the beginning of each experiment, *O_b_* ≡ *O_n_*. Moreover, the origin of {**s**} *O_s_* was where the barometric altimeter is located within the IMU case, which is actually very close to where the tri-axial accelerometer is also located.

In this paper, {**s**} and {**b**} were coincident, except when the sensor unit was submitted to conditions of forced circular motion: in the latter case the orientation matrix from {**s**} to {**b**} was the identity matrix (at first approximation), while *O_s_* was displaced relative to *O_b_* by the lever-arm vector **L**.

**Figure 2.**
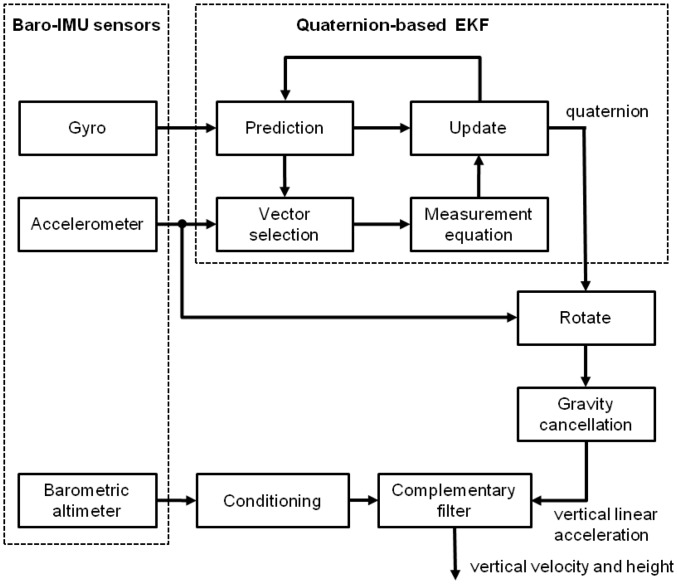
Block diagram of the sensor fusion method for estimating vertical velocity and height using baro-IMU sensor data.

Figure 2 shows the block diagram of the developed sensor fusion method. Two filtering modules were cascaded: a filtering module (the quaternion-based EKF) computed the attitude of {**b**} relative to {**n**} using the inertial sensors of the sensor unit. The linear acceleration was then estimated by rotating the specific force measured by the tri-axial accelerometer from {**b**} to {**n**} and adding the gravity to it (gravity cancellation). The vertical component of the linear acceleration and the output from the barometric altimeter were then fed into the complementary filter, where estimates of the vertical velocity and height were finally produced and resolved in {**n**}.

#### 2.1.1. Quaternion-Based EKF for Attitude Estimation

The filtering algorithm for estimating the attitude of {**b**} relative to {**n**} is a modified version of the quaternion-based EKF developed in [21]. In the present implementation, the state vector **x** includes the quaternion **q̄**^*nb*^ and the bias vector *^a^***b** for self-compensation of accelerometer biases developing over time, for the statistical description of which a first-order Gauss-Markov model is assumed [22]. According to this model, *^a^***b** is the realization of an exponentially time-correlated vector stochastic process whose components are statistically independent:

(1) b˙a=−aba+va,

where *α* is the reciprocal of the correlation time constant τ, and *^a^***v** is white Gaussian noise, with zero mean and covariance matrix *^a^***Σ = I**_3×3_ · *^a^σ*^2^ (**I***_n_*_×_*_n_* is the *n* × *n* identity matrix).

The quaternion kinematics equation can be written as follows:

(2) $$\frac{d}{dt}{\bar{\text{q}}}^{nb}=f\left({\bar{\text{q}}}^{nb},t\right)=\frac{1}{2}\text{Ω}\left(\omega_{nb}^{b}\left(t\right)\right){\bar{\text{q}}}^{nb}$$

where:

(3) $$\text{Ω}\left({\text{ω}}_{nb}^{b}\right)=\left[\begin{array}{cc} -\left[{\text{ω}}_{nb}^{b}\times \right] & {\text{ω}}_{nb}^{b}\\ -{\left({\text{ω}}_{nb}^{b}\right)}^{T} & 0 \end{array}\right]$$

${\text{ω}}_{nb}^{b}={\left[\begin{array}{ccc} p & q & r \end{array}\right]}^{T}$ is the angular velocity of {**b**} relative to {**n**} resolved in {**b**}, and:

(4) $$\left[{\text{ω}}_{nb}^{b}\times \right]=\left[\begin{array}{ccc} 0 & -r & q\\ r & 0 & -p\\ -q & p & 0 \end{array}\right]$$

is the skew-symmetric cross-product matrix.

We consider the following discrete-time process of the quaternion kinematics of Equation (2):

(5) $${\bar{\text{q}}}_{k}^{nb}=\Phi_{k-1}{\bar{\text{q}}}_{k-1}^{nb}=\exp\left(\frac{T_{s}}{2}\text{Ω}\left({\text{ω}}_{nb}^{b}\left(k-1\right)\right)\right){\bar{\text{q}}}_{k-1}^{nb}$$

where *T_s_* is the sampling interval. Moreover, the discrete-time model of Equation (1) can be written as follows:

(6) bak=I3×3·exp(−αTs)bak−1+wak−1

where the covariance matrix of the process noise *^a^***w***_k_*_−1_ is given by:

(7) Qa=I3×3·1−exp(−2αTs)2ασa2

The state transition equation is given by:

(8) [q¯knbbak]=Fk−1[q¯k−1nbbak−1]+[wqk−1wak−1]=[Φk−103×303×3I3×3·exp(−αTs)][q¯k−1nbbak−1]+[wqk−1wak−1]

The gyro measurement noise **v***_g_* is white Gaussian noise with zero mean and covariance matrix $\sum_{g}={\text{I}}_{3\times 3}\cdot \sigma_{g}^{2}$. The process noise component *^q^***w***_k_*_−1_ describes how **v***_g_* enters the state model of Equation (5) through the following quaternion-dependent linear transformation:

(9) wqk−1=−Ts2Ξ(q¯k−1nb)vgk−1

and the matrix **Ξ**(**q̄**) is given by:

(10) $$\Xi \left(\bar{\text{q}}\right)=\left[\begin{array}{c} {\text{I}}_{3\times 3}\cdot q_{4}+\left[\text{q}\times \right]\\ -{\text{q}}^{T} \end{array}\right]$$

Since the process noise components *^q^***w***_k_*_−1_ and *^a^***w***_k_*_−1_ are assumed to be uncorrelated, the process noise covariance matrix **Q***_k_*_−1_ has the following structure:

(11) Qk−1[(I4×4·trace(M)−M)·σg2(Ts2)203×303×3Qa]

where:

(12) $$\text{M}={\bar{\text{q}}}^{n\text{b}}\left(k|k-1\right){\left({\bar{\text{q}}}^{n\text{b}}\left(k|k-1\right)\right)}^{T}+{\text{P}}^{q}\left(k|k-1\right)$$

${\bar{q}}^{nb}\left(k|k-1\right)$ and **P***^q^* (*k*|*k* − 1) denote, respectively, the expectation and the error state covariance matrix of the quaternion component of the state vector after propagation through Equation (8) [23]:

(13) $$\begin{array}{l} \text{x}\left(k|k-1\right)={\text{F}}_{k-1}\text{x}\left(k-1|k-1\right)\\ \text{P}\left(k|k-1\right)={\text{F}}_{k-1}\text{P}\left(k-1|k-1\right){\text{F}}_{k-1}^{T}+{\text{Q}}_{k-1} \end{array}$$

We remember that the propagated state is denoted as **x**(*k*|*k* − 1), to mean that the accelerometer information available to the Kalman-based filter at time *kT_s_* is still to be used for computing **x**(*k*|*k*), (update step).

The specific force measured by the tri-axial accelerometer has the following expression (in the {**b**}-frame) [3]:

(14) $${\text{f}}^{b}={\text{C}}^{bn}\left({\ddot{\text{b}}}^{n}-{\text{g}}^{n}\right)+\left[{\dot{\text{ω}}}_{nb}^{b}\times \right]{\text{s}}^{b}+\left[{\text{ω}}_{nb}^{b}\times \right]\;\left[{\text{ω}}_{nb}^{b}\times \right]{\text{s}}^{b},$$

where:

(15) $${\text{C}}^{bn}={\text{I}}_{3\times 3}+2q_{4}^{nb}\left[{\text{q}}^{nb}\times \right]+2{\left[{\text{q}}^{nb}\times \right]}^{2}.$$

In Equation (14) **g***^n^* is the gravity acceleration resolved in {**n**}, $\left[{\dot{\text{ω}}}_{nb}^{b}\times \right]{\text{s}}^{b}$ and $\left[{\text{ω}}_{nb}^{b}\times \right]\;\left[{\text{ω}}_{nb}^{b}\times \right]{\text{s}}^{b}$ are the tangential and centripetal acceleration, respectively. Equation (14) is valid under the assumption that the Coriolis acceleration is negligible.

Accelerometer leveling is used to determine inclination, according to the following measurement equation:

(16) zk=h(q¯knb,bak)=Cbn(q¯knb)(−gn)+bak+vfk,

where the measurement noise **v***_f k_* is white Gaussian noise with zero mean and covariance matrix $\text{R}={\text{I}}_{3\times 3}\cdot \sigma_{a}^{2}$. Accelerometer leveling is potentially undermined by the presence of non-ravitational components of acceleration, which include the linear acceleration **b̈^*n*^** and the components due to any rotational motion of the {**s**}-frame relative to the {**n**}-frame.

Only for slow tracked motions, or when the lever-arm length is null, the specific force measurements can be safely used for state update in the EKF. Because of this, vector selection methods are usually employed to guard against the effects of fast motions. The method implemented in this paper looks at the innovation produced within the filter:

(17) vk=fkb−Cbn(q¯nb(k|k−1))(−gn)−ba(k|k−1).

Anytime components of the innovation vector exceed in magnitude a properly specified threshold value *λ_g_* their rejection from the update step is obtained by setting the corresponding rows of the EKF measurement matrix **H** to zero:

(18) H(q¯nb,ba)=∂z∂x=[−2q4nb[gn×]−2([gn×][qnb×]−2[qnb×][gn×])2[qnb×]gnI3×3],

where use is made of ${\bar{q}}^{nb}\left(k|k-1\right)$ for computation. Let **H***_a_* be the matrix resulting from this procedure of row-wise cancellation.

The Kalman gain is computed according to the following equation:

(19) $$\text{K}=\text{P}\left(k|k-1\right){\text{H}}_{a}^{T}{\left({\text{H}}_{a}\text{P}\left(k|k-1\right){\text{H}}_{a}^{T}+\text{R}\right)}^{-1}.$$

The update of the state vector and error state covariance matrix is given by:

(20) $$\begin{array}{l} \text{x}\left(k|k\right)=\text{x}\left(k|k-1\right)+\text{K}{\text{v}}_{k}\\ \text{P}\left(k|k\right)=\left(\text{I}-{\text{KH}}_{a}\right)\text{P}\left(k|k-1\right). \end{array}$$

In order for the quaternion component of the state vector to represent a valid rotation, it is standard practice to submit the updated quaternion to brute-force normalization in preparation for the next iteration [23]:

(21) $${\bar{\text{q}}}^{nb}\left(k|k\right)=\frac{{\bar{\text{q}}}^{nb}\left(k|k\right)}{\left\|{\bar{\text{q}}}^{nb}\left(k|k\right)\right\|}$$

#### 2.1.2. Complementary Filter for Vertical Velocity and Height Estimation

To get the linear acceleration the specific force must be rotated from {**b**} to {**n**}:

(22) $${\ddot{\text{x}}}_{a\;k}={\text{C}}^{nb}{\text{f}}_{k}^{b}+{\text{g}}^{n},$$

where the unit-norm quaternion ${\bar{q}}^{nb}\left(k|k\right)$ is plugged into the expression of the rotation matrix:

(23) $${\text{C}}^{nb}={\text{I}}_{3\times 3}-2q_{4}^{nb}\left[{\text{q}}^{nb}\times \right]+2{\left[{\text{q}}^{nb}\times \right]}^{2}.$$

Upon rotation and gravity compensation, **ẍ _*a k*_** can be submitted to single- and double-time integration to estimate velocity and position, respectively. This is the classical strap-down approach to inertial navigation [24]. Horizontal positioning, for which accurate heading estimation is also needed [25], is outside the scope of this paper. Here, only the vertical component *ẍ _a k_* of the linear acceleration **ẍ _*a k*_** is retained for further processing.

The complementary filter can be implemented as suggested in [19]. The system's state *^c^***x** includes two variables, the vertical position and the vertical velocity:

(24) xck=[xakx˙ak]=[1Ts01]xck−1+[1Ts/201]Kc·TsΔxk−1+[Ts/21]Δυk−1,

where Δ*x_k_* = *x_p k_* − *x_a k_* and Δ*v_k_* = *T_s_ẍ_a k_* ·*x_p k_* is the pressure altitude measured by the barometric altimeter at time *t_k_* = *kT_s_* and *ẍ _a k_* is the vertical linear acceleration from Equation (22) at the same time instant.

The gain **K***_c_* of the complementary filter is given by:

(25) $${\text{K}}_{c}=-\left[\begin{array}{c} \sqrt{2\sigma_{w}/\sigma_{v}}\\ \sigma_{w}/\sigma_{v} \end{array}\right]$$

where *σ_w_* and *σ_v_* are the standard deviations of the noise in the linear acceleration *ẍ _a k_* and in the pressure altitude *x_p k_*, respectively. The complementary filter has low-pass characteristics with time constant:

(26) $$\tau =\sqrt{\frac{\sigma_{v}}{\sigma_{w}}}$$

The assumptions for the validity of the set of difference Equation (24) and optimality of the gain Equation (25) are the following: (a) the sampling interval is small enough that Δ*x_k_* and *ẍ _a k_* can be taken constant over the sampling interval; (b) the noise in the linear acceleration estimate and the noise in the pressure altitude measurement are mutually uncorrelated zero-mean white Gaussian noises.

Short-time correlated fluctuations due to environment dependent pressure changes are present in the output signal from motionless barometric altimeters. It is this serial correlation that reduces the efficiency of, e.g., *M*-point moving average filters in smoothing noisy data from barometric altimeters. Recently, we have developed a stochastic method for barometric altimeter noise modeling using autoregressive moving average (ARMA) identification techniques [20]. The method was successful in identifying the short-time correlated noise component, whose effects are prominent for short-time tracking. Our idea was to use the learnt ARMA model parameters to design a whitening filter that would remove the serial correlation from the measured pressure altitude; for the experiments in this paper the whitening filter was designed with a DC gain *A*_DC_ = 0.21, one pole at a frequency of about 1 Hz and one zero approximately two octave down. Accordingly, a unity-gain constraint was enforced at 25 Hz. In the block named Conditioning in Figure 2, we applied either a 4-point moving average filter (Method A) or a 4-point moving average filter cascaded with the whitening filter (Method B), and tested their effect on the tracking accuracy.

### 2.2. Sensor Hardware

The experiments described in this paper were performed using a battery-powered wearable inertial measurement unit named Wearable Inertial Measurement Unit (WIMU), whose development is underway in our lab for applications in human motion ambulatory monitoring and assessment. The WIMU is endowed with a 32-bit ARM Cortex processor (NXP Semiconductors LPC1768) and a Bluetooth (BT) transceiver, which is currently implemented in a version for data communication with an Android-based smart-phone (Samsung Galaxy SII, GT-I9100). The WIMU sensors are a digital tri-axial gyro (InvenSense ITG-3200), a digital tri-axial accelerometer (Bosch BMA180), a digital tri-axial magnetic sensor (Honeywell HMC5843) and a digital pressure sensor (Bosch BMP085).

Two wireless nodes, *i.e.*, the smart-phone and a PC running MATLAB, communicated in a peer-to-peer mode within a Wireless Local Area Network (WLAN) via a User Datagram Protocol (UDP) connection, which allowed upload of 22-bytes sensor data packets at each sample time in the MATLAB workspace. The sensor fusion method was written and implemented in MATLAB to work in online conditions at the rate of 50 Hz. It is noted that, in order to perform gravity compensation, the specific force measured by the tri-axial accelerometer has to be projected along the vertical direction; heading estimation is not necessary to implement this procedure, hence our decision was not to use the magnetic sensor measurements available from the WIMU.

The measurements by the BMP085 digital pressure sensor are noisy, including a significant amount of quantization noise due to a coarse resolution of about 1 Pa (corresponding to about 8.43 cm). The BMP085 was used in the ultra-low power mode (no oversampling was performed internally to the sensor). The ANSI C code by the sensor manufacturer was used to implement the compensation algorithm for pressure and temperature measurement using the integrated thermal sensor. The temperature was measured once per second, and this value was used for all pressure altitude measurements during the same period. The ANSI C code was ported to the ARM controller, together with the routine for calculating the altitude from calibrated pressure measurements using the barometric formula:

(27) $$h=44300\left(1-{\left(\frac{p}{p_{o}}\right)}^{0.19}\right)$$

where *h* is expressed in m and *p_o_* = 1013.25 hPa is the standard pressure at sea level.

Absolute altitude information cannot be easily obtained by barometric altimeters. Equation (27) still applies in cases where the pressure and the temperature differ from those of the standard: the relative change in pressure and the actual temperature determine the change in altitude regardless of altitude. In all our experiments the relative pressure altitude was computed by taking the average value of the absolute pressure altitude during a rest period of 1 s before the motion started; the absolute pressure altitude signals during each experimental trial were then detrended by subtracting this constant value from them in the Conditioning block of Figure 2 (bias capture), yielding the height relative to the baro-IMU initial position.

### 2.3. Experimental Validation

The experimental validation of the proposed methods was based on submitting the baro-IMU to different motion conditions, namely: No-motion, free-fall motion, forced circular motion and squatting. The motion conditions were tested by repeating each trial *N* times (*N* = 10). When reference data were available (*i.e.*, no-motion, free-fall motion, forced circular motion), Root Mean Square values of the difference (Error) between estimated (*y*_est_) and reference (*y*_ref_) time functions of the variables of interest (*i.e.*, height and vertical velocity) were computed for each trial, motion condition and sensor fusion method. For instance, in the case of no-motion and forced circular motion conditions we have:

(28) $$\text{RMSE}=\sqrt{\frac{1}{S}\sum_{k=1}^{S}{\left(y_{\text{est}}\left(k\right)-y_{\text{ref}}\left(k\right)\right)}^{2}}$$

where *S* was the number of samples available in each trial (*S* = 9000). As for the free-fall motion condition, the reference time functions were based on a free-fall model and were compared to estimated time functions of the variables of interest during the fall, see Section 2.3.2 for details.

The Bartlett test for equal variance was applied to RMSE values of each variable of interest produced by Method A and Method B in each motion condition (5% significance level). Finally, the paired-sample Wilcoxon signed rank test was applied to assess the RMSE performance between the two methods.

#### 2.3.1. No-Motion

The experiment consisted of taking the baro-IMU at rest on top of a table while sensor data were acquired in a time interval lasting three minutes. The aim was to assess the effects of the short-time correlated pressure altitude fluctuations in the barometric altimeter output on the accuracy of height and vertical velocity estimation. Since the baro-IMU was motionless, the reference height and velocity to be entered in the RMSE calculation were null.

#### 2.3.2. Free-Fall Motion

The free-fall motion consisted of letting the baro-IMU fall from a known height *H* onto a soft mattress. The baro-IMU was initially at rest on a shelf at the height of 1.53 m above the mattress (rest phase I). The experimenter gently grasped the baro-IMU by hand and displaced it laterally trying to avoid any height change (rest phase II). The experimenter then released the baro-IMU, which fell down until the first impact on the mattress took place (free-fall phase I). No care was taken in order that the orientation of the sensor case was fixed during the fall. Eventually the baro-IMU rebounded to fall down again during the free-fall phase II. Finally, the baro-IMU went to rest on the mattress (rest phase III).

During a free-fall motion the inertial frame vertical acceleration is equal to the gravity acceleration *g* (*g* = 9.81·m/s^2^). Under the simplifying assumption of zero air resistance, the vertical velocity and position are therefore given by:

(29) $$\{\begin{array}{l} v\left(t\right)=gt\\ h\left(t\right)=\frac{1}{2}gt^{2} \end{array}$$

for *t* in the interval [0, *T*_fall_], where the impact time *T*_fall_ is:

(30) $$T_{\text{fall}}=\sqrt{\frac{2H}{g}}$$

Equation (29) was used to compute the reference time functions against which to compare the estimated height and vertical velocity during the free-fall phase prior to the first impact for RMSE calculation. Since the {**s**} and {**b**} frames were coincident in this case, the specific force in Equation (14) was zero during the free-fall phase:

(31) $${\text{f}}^{b}={\text{C}}^{bn}\left({\ddot{\text{b}}}^{n}-{\text{g}}^{n}\right)={\text{C}}^{bn}\left({\text{g}}^{n}-{\text{g}}^{n}\right)=\text{0}.$$

#### 2.3.3. Forced Circular Motion

The baro-IMU was submitted to a nominally uniform circular motion. For carrying this experiment we used a mechanical platform consisting of a DC motor (3863A024C Faulhaber) with its axis of rotation perpendicular to gravity. A plastic rod was hinged on the motor axis and the baro-IMU was fastened to the rod at a distance of *L* = 30 cm from the motor axis, with the axes of {**s**} parallel to the axes of {**b**}. The DC motor was controlled using a motion controller (MCDC3006S Faulhaber) that was interfaced to a PC via a standard RS232 port, yielding measured angular displacements relative to a zero position with the axes of {**s**} and {**b**} aligned with the axes of {**n**}. The origins of {**n**} and {**b**} were coincident at all times; the origin of {**s**} was shifted along the plastic rod by the lever arm *L*.

Angular displacements were measured using the built-in incremental encoder and were delivered to the PC at a rate of 250 Hz (*T* = 4 ms). A Graphical User Interface (GUI) was written in MS Visual Basic 6.0 to allow the experimenter to set the speed of rotation at a given value *ω_o_* = 2*πf_o_*. Synchronization between baro-IMU sensor and encoder signals was achieved by looking for the time instant when the measured angular rate exceeded a threshold *λ* in both signals (*λ* = 5 °/s); encoder data alignment was performed using cubic interpolation.

In our experimental set-up we have:

(32) $$\{\begin{array}{l} {\text{s}}^{b}={\left[\begin{array}{ccc} 0 & L & 0 \end{array}\right]}^{T}\\ {\ddot{\text{b}}}^{n}=0\\ {\text{ω}}_{nb}^{b}={\left[\begin{array}{ccc} \omega_{o} & 0 & 0 \end{array}\right]}^{T} \end{array}$$

In the case of a uniform circular motion, Equation (14) can be written:

(33) $${\text{f}}^{b}=\left[\begin{array}{ccc} 1 & 0 & 0\\ 0 & \cos\omega_{o}t & \sin\omega_{o}t\\ 0 & -\sin\omega_{o}t & \cos\omega_{o}t \end{array}\right]\;\left[\begin{array}{c} 0\\ 0\\ -g \end{array}\right]+\left[\begin{array}{c} 0\\ -\omega_{o}^{2}L\\ 0 \end{array}\right]=\left[\begin{array}{c} 0\\ -g\sin\omega_{o}t-\omega_{o}^{2}L\\ -g\cos\omega_{o}t \end{array}\right]$$

The linear acceleration of the origin of {**s**} resolved in {**n**} is thus given by:

(34) $${\ddot{\text{s}}}^{n}=\left[\begin{array}{ccc} 1 & 0 & 0\\ 0 & \cos\omega_{o}t & -\sin\omega_{o}t\\ 0 & \sin\omega_{o}t & \cos\omega_{o}t \end{array}\right]\;\left[\begin{array}{c} 0\\ -g\sin\omega_{o}t-\omega_{o}^{2}L\\ -g\cos\omega_{o}t \end{array}\right]+\left[\begin{array}{c} 0\\ 0\\ g \end{array}\right]=\left[\begin{array}{c} 0\\ -\omega_{o}^{2}L\cos\omega_{o}t\\ -\omega_{o}^{2}L\sin\omega_{o}t \end{array}\right]$$

yielding a harmonic motion law with displacement-time function:

(35) $${\text{s}}^{n}=\left[\begin{array}{c} 0\\ L\cos\omega_{o}t\\ L\sin\omega_{o}t \end{array}\right]$$

The baro-IMU height relative to its initial position was estimated using the interpolated angular displacements *θ_k_* from the incremental encoder and the known lever-arm length *L*:

(36) $$h_{k}=L\sin\theta_{k}$$

Finally, a central finite difference approximation was used to estimate the vertical velocity from the estimated height:

(37) $${\dot{h}}_{k}=\frac{h_{k-1}-h_{k-1}}{2T_{s}}$$

The computed height and vertical velocity were used as reference data for RMSE calculation. 3-min long trials were performed once for five values of motion frequency *f_o_*.

#### 2.3.4. Squatting

The final experiment was carried out as follows: a subject wore the baro-IMU at the upper trunk level. Standing still from the upright posture, he was asked to perform a squat before coming to the initial posture. In this case, reference height and vertical velocity for RMSE calculation were not available; for comparative purposes, the method of vertical linear velocity estimation using inertial sensors described in [26], henceforth called the reference method, was implemented.

Briefly, the reference method works as follows: the tri-axial accelerometer is used during periods of quasi-static activity to provide the initial conditions of orientation for the rotational mapping of the gravity vector through strap-down integration of the angular velocity during periods of dynamic activity. Vertical linear acceleration during periods of dynamic activity is then integrated. Because of integration drift errors, initial and end conditions of each period of dynamic activity, in terms of both the orientation and the vertical linear velocity, may differ from one another. The orientation computed using the tri-axial accelerometer and the nominally null linear velocity during the periods of quasi-static activity are used for estimating and correcting the integration drift error [26].

### 2.4. Implementation Details

The initial orientation of {**b**} relative to {**n**} was computed by leaving the baro-IMU motionless for a rest period of about 1 s before starting to move. For instance, in the case of the squatting exercise, the subject was instructed to stand still before performing the squat. Initial conditions for the complementary filter were zero, either in height or velocity.

Gyro bias was estimated by taking the average of the tri-axial gyro output during the rest period, which was then removed from the measured angular rates *p, q, r* before running the sensor fusion method (bias capture) [18]. The in-field calibration of the tri-axial accelerometer was performed using the approach described in [27].

After applying the sensor fusion method to the datasets available for each experimental condition described in this paper, the values of the EKF parameters that we found appropriate for its use are reported in Table 1.

The different values of the barometric altimeter noise standard deviation *σ_v_* depended on which filtering method was applied in the conditioning block (the smallest value was chosen in the case of Method B). Due to minute motions of the body where it was affixed, the *apparent* noisiness of the accelerometer was higher than in truly motionless conditions, hence the value of the standard deviation *σ_a_* was higher than values expected from bench calibration (for a typical MEMS accelerometer, 1–2 m*g*) [18]. The standard deviation *σ_w_* of the noise in the linear acceleration *measurements* was further increased to account for additional errors that may arise in, e.g., the strap-down rotation.

In the present implementation, 9 ms were needed (on average) to run each iteration step of the sensor fusion method (EKF cycle of prediction and update, followed by strap-down rotation and complementary filtering), which included about 6 ms consumed for uploading the 22-bytes sensor data packets available at each sample time to the MATLAB workspace.

## 3. Results

### 3.1. No-Motion

The RMSE values of height and vertical velocity were computed using the 9000 samples collected during the 3 min-long trials for each sensor fusion method, Table 2. In this and the following tables, asterisks indicate statistical significances of the paired-sample Wilcoxon signed rank test as follows: * *p* < 0.05, ** *p* < 0.01. The Bartlett test rejected the null hypothesis of equal variance of the height RMSE values produced by the two methods at the significance level 5%. We can conclude that Method B outperformed Method A in this motion condition.

**Figure 3.**
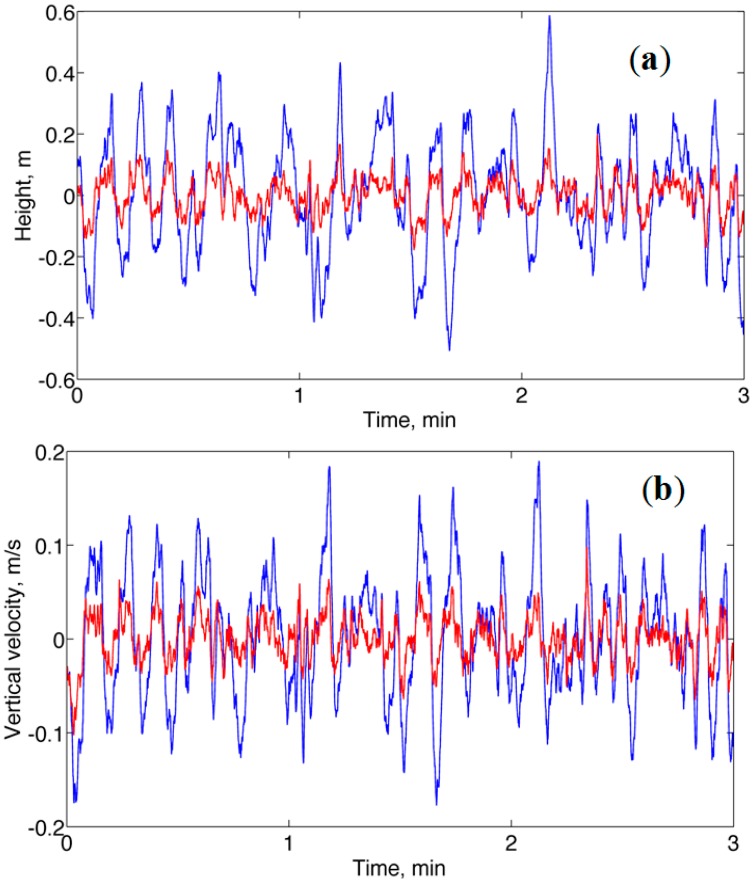
No-motion. (**a**) Height time functions are plotted in blue (Method A) and red (Method B); (**b**) Vertical velocity time functions are plotted in blue (Method A) and red (Method B). The reference height and vertical velocity are zero.

A representative plot the height and vertical velocity time functions estimated with either method A or method B is given in Figure 3. It is noted that the vertical velocity estimates were drift-free, in a situation where the vertical linear acceleration estimates were substantially zero mean (the mean of vertical linear acceleration was about 2 m*g*, with standard deviation SD of about 2 m*g*). The bias *B* affecting the estimates of vertical velocity was about 0.01 m/s, on average, for either Method A or Method B).

### 3.2. Free-Fall Motion

For RMSE calculation the experimental time functions were compared with Equation (20) over the time interval [*T*_start_ *T*_end_]; by our definition, *T*_start_ was the time instant when the norm of the specific force came to be equal to *g*/2 during the free-fall phase I, while *T*_end_ was computed by adding the expression of Equation (30) to *T*_start_. The results of the RMSE analysis are reported in Table 3. In this motion condition the Bartlett test and the paired-sample Wilcoxon signed rank test failed to reach statistical significance, thus implying that the two methods were nearly equivalent.

**Figure 4.**
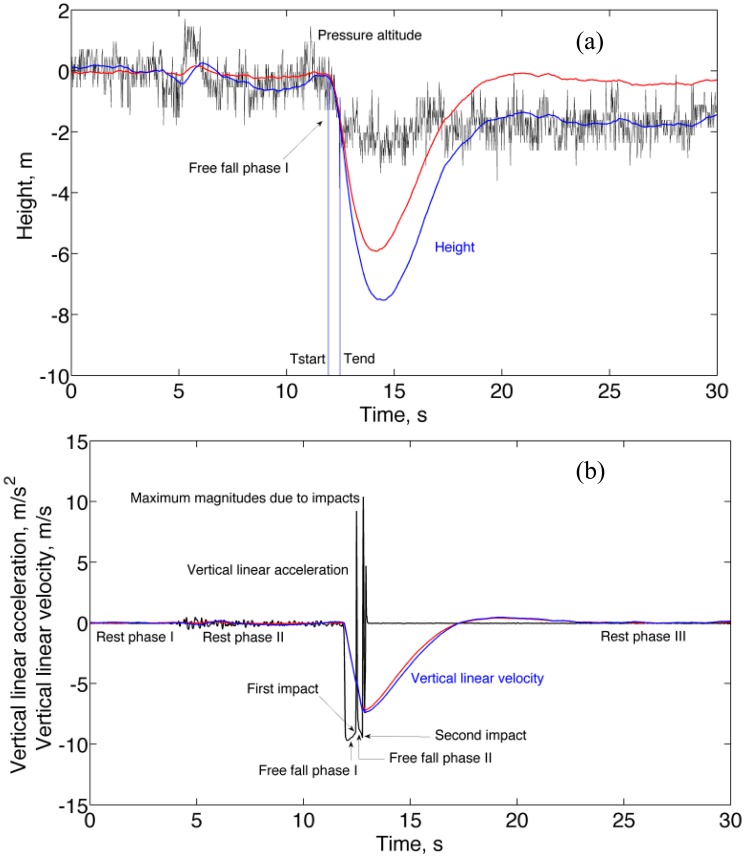
Free-fall motion. (**a**) Height estimated by Method A (blue) and Method B (red) with superimposed the raw pressure altitude (black); (**b**) Vertical velocity estimated by Method A (blue) and Method B (red) with superimposed the vertical linear acceleration (black).

A representative plot of height and vertical velocity estimated using either Method A or Method B is given in Figure 4. As shown in Figure 4, the rebound of the WIMU took place during the acceleration signal hump observed at the end of each free-fall phase; at the end of the free-fall phase I the vertical velocity would change sign, before reverting again to the linear trend observed during the succeeding free-fall phase II. However, the velocity state at the output of the complementary filter does not change sign during the impact-related acceleration signal humps. The reason for the estimation errors growing so large after the first impact is unclear: sensor saturation was not observed in our experiments, maybe limitations in sensor bandwidth play a role. The height and vertical velocity estimated by the complementary filter after impacts were thus heavily biased downwards, and it took several seconds before the complementary filter recovered during the rest phase III. The recovery period after the first impact was about 8 s, consistent with the value of the time constant of the complementary filter—see Equation (26). Actually, the behavior of the vertical velocity during the first rebound phase was very similar to the one showed in [28], where the tracking exercise came apparently to end just after the first impact.

It is also noted that, in contrast with Method A, Method B was unable to accurately track the height during rest phase III. The reason is that the whitening filter may distort the motion-related low-frequency components in the signal produced by the barometric altimeter. In particular, the whitening filter adopted in this work has a DC gain of 0.21, which explains why the height was underestimated by Method B during rest phase III.

### 3.3. Forced Circular Motion

The RMSE values of height and vertical velocity were computed using the 9000 samples collected during the 3 min-long trials for each value of motion frequency *f_o_* and sensor fusion method, Table 4. The Bartlett test reached statistical significance for height RMSE, while the paired-sample Wilcoxon signed rank test failed to reach statistical significance only for the height RMSE when *f_o_* = 1.25 Hz, in which case both methods performed poorly.

**Figure 5.**
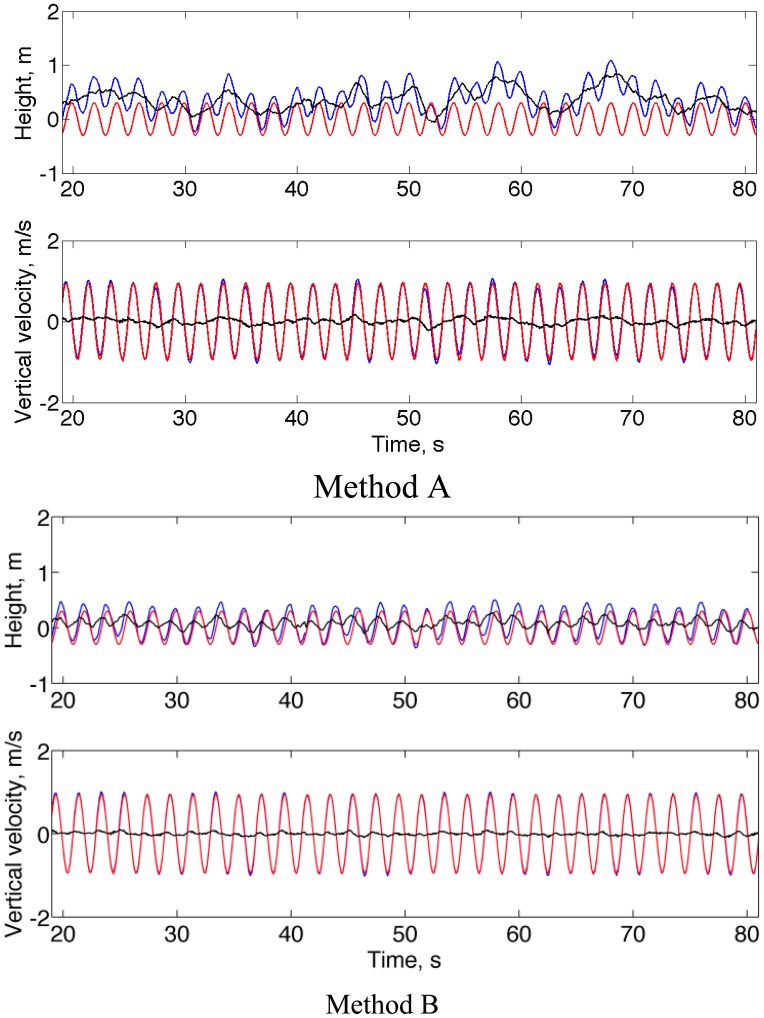
Forced circular motion. Estimated (blue) and reference (red) height and vertical velocity time functions, with superimposed the difference error between estimated and reference time functions (black). Data are displayed in the time interval from 20 to 80 s for improved graphical readability.

Figure 5 shows representative plots of the estimated height and vertical velocity when the motion frequency is 0.5 Hz. As in the no-motion condition, it is noted that Method B outperformed Method A.

### 3.4. Squatting

**Figure 6.**
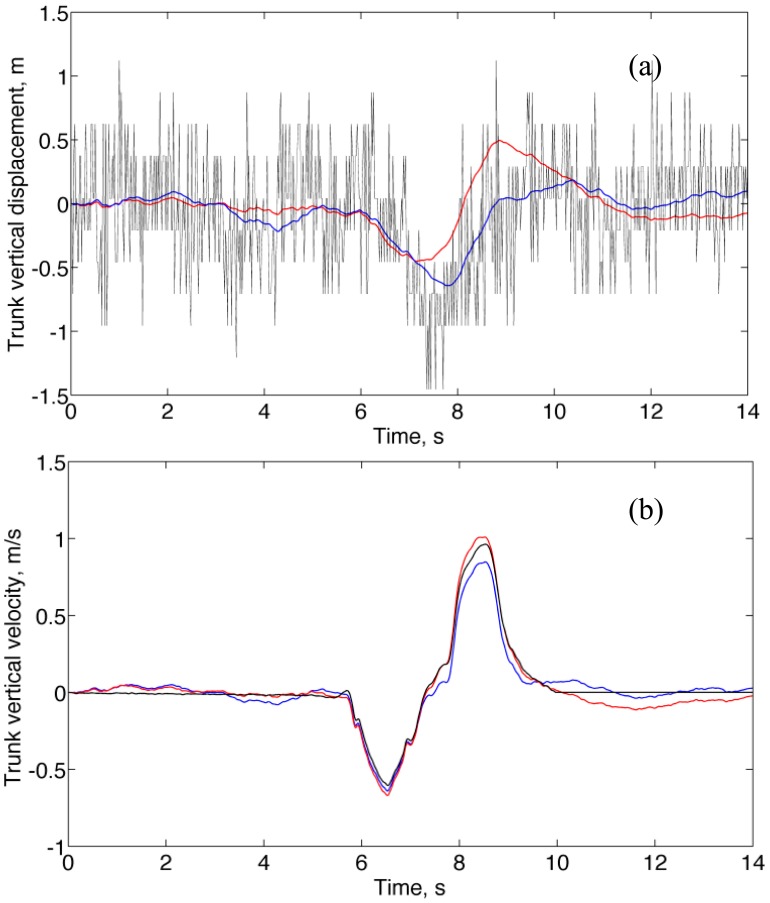
Squatting motion. (**a**) Trunk vertical displacement estimated by Method A (blue) and Method B (red) with superimposed the raw pressure altitude (black); (**b**) Trunk vertical velocity estimated by Method A (blue) and Method B (red), with superimposed the estimate by the reference method (black).

Figure 6a shows a representative plot of the trunk vertical displacement estimated by either Method A or Method B with superimposed the raw data from the barometric altimeter. The trunk vertical velocity estimated by Method A, Method B and the reference method are plotted in Figure 6b for the same squat exercise as in Figure 6a. The maximum trunk vertical displacement from standing still to squat position was measured by hand (approximately, *H* = 0.60 m); the estimates produced by the sensor fusion methods were 0.86 ± 0.39 m (Method A) and 0.50 ± 0.11 (Method B). According to the results of the Bartlett test, the maximum trunk vertical displacement estimated by Method A had a higher variance compared with Method B. Moreover, a one-sample Wilcoxon signed rank test that the estimated maximum trunk vertical displacement comes from a distribution with median *H* failed at the 5% significance level for Method B. It is noted that, in contrast with Method A, Method B tended to underestimate the maximum trunk vertical displacement. The RMSE of the trunk vertical velocity was 0.14 ± 0.10 for both methods.

## 4. Discussion

One interesting point of discussion common to all experimental conditions in this paper is that the estimate of the vertical velocity is virtually drift-free. This is not obviously the case when vertical linear acceleration is submitted to single-time integration.

Suppose that the accelerometer works without scale or offset errors; additionally, let us assume that the strap-down rotation is implemented without errors. Even under these ideal conditions, integration of *noisy* acceleration data is not without errors: the variance of random-walk integration errors grows linearly with integration time and is proportional to the accelerometer measurement noise variance [3]. On the other hand, any uncompensated scale and offset errors give rise to linearly drifting velocity estimates; moreover, orientation errors are known to affect the accuracy of gravity cancellation to a great extent [29,30]. An interesting discussion concerning the effects of sensor and orientation errors on the accuracy of strap-down integration is reported in [31]. There, handling the nonlinearity involved in implementing the strap-down rotation is referred to as the umbrella problem. The probability density function of the rotated acceleration is not indeed a Gaussian shape, but looks like an umbrella, yielding a downward bias in the vertical linear acceleration estimate. Higher-order EKFs, namely the second-order EKFs investigated in [5] for their capability of modeling the nonlinearity involved in the strap-down rotation, were not considered for implementation at the current stage of our research. The bias error cannot be negligible when large orientation errors occur or when large horizontal accelerations are present.

Orientation errors can grow large especially when fast motions are to be tracked. In particular, this difficulty may be due to limitations in the sampling rates, or the low reliability of accelerometer leveling. The free-fall and forced circular motion experiments were particularly challenging as for the reliability of leveling. In the case of free-fall motion, all specific force measurements were rejected by the vector selection method during the free-fall phases, when the specific force was nominally zero. Hence quaternion propagation and update were based only on the gyro measurements. In the case of forced circular motion, and especially with increases in the values of the frequency of motion, the proposed vector selection method rejected a progressively large proportion of specific force measurements along the Y-axis, which were dominated by the centripetal acceleration components; the specific force measurements along the other sensitivity axes passed the test and were thus used for leveling.

It is noted that the height and vertical velocity RMSE values tended to increase for the highest values of the forced circular motion frequency, Table 4, where we observed a slight downward bias of few m*g*'s in the vertical linear acceleration – likely, an instance of the umbrella problem. Empirically, the accelerometer-bias compensation was instrumental in reducing the downward bias of the vertical linear acceleration, in line with the notion that adding a limited amount of pseudo-noise to components of the state vector may stabilize Kalman-based filters [32]. In our previous work, we demonstrated that gyro-bias compensation can help increasing the accuracy of orientation estimation according to the same principle of pseudo-noise injection, even when gyro-bias is not given time to change significantly, namely when the duration of the tracked motion does not exceed, say, few minutes [18]. Although it should be feasible and technically straightforward, we decided not to implement the gyro-bias compensation at the present stage of system development.

An attractive feature of the complementary filter was that, thanks to the contribution of the barometric altimeter, drifts in the estimated variables of interest could be virtually eliminated, in the vertical velocity domain at least. The height and vertical velocity tracking accuracy of the complementary filter increased when the whitening filter was applied in the conditioning block in the case of no-motion and forced circular motion. This is due to the whitening filter acting to make the barometric altimeter noise white, a requisite for which the complementary filter is optimal [19]. However, the whitening filter introduced linear distortions of amplitude and phase on the pressure altitude changes that were due to sensor motion. These distortions can explain the fact that, in contrast with Method A, Method B was unable to track accurately the height during the rest phase III in Figure 4a, and underestimated the maximum trunk vertical displacement in Figure 6a.

One problem with using the whitening filter is related to where the unity-gain constraint is enforced. Since the correlated noise component occupies the low-frequency part of the spectrum, the whitening filter presents a typically high-pass frequency response, which tends to amplify uncorrelated noise unless the unity-gain constraint is moved from DC to higher frequencies. On the one hand, based on the ARMA model identification results, the −3 dB corner frequency of the whitening filter is approximately 1 Hz, which makes distortionless tracking quite difficult for slow human motions in the vertical direction. On the other hand, the amplifying effect on the uncorrelated noise component due to a unity-gain constraint enforced at DC would require, approximately, a 25-fold increase in the length of the *M*-point moving average filter for achieving the same Signal-to-Noise ratio, which is unacceptable due to the amplitude and phase distortions introduced in the filtering chain. We conclude that, because of their limited measurement capabilities, barometric altimeters are critical components for doing accurate relative height tracking of human-like motions. It is believed that technological advancements may improve the picture in the near future [17]. For now, we have demonstrated that, although the whitening filter does not allow improving the tracking accuracy in free-fall and squatting conditions, the vertical velocity is estimated fairly well with both methods, even in comparison with the reference method, which is quoted to have a vertical velocity RMSE in the order of 0.1 m/s against ground-truth data from an optoelectronic motion analysis system [26]. Usually, when dealing with inertial sensing applications, dedrifting algorithms such as those implemented in the reference method are applied to estimates of velocity that are produced by time-integration of rotated acceleration data to improve accuracy [23,26]. By their nature, these methods work off-line, while the proposed sensor fusion method works on-line. It is worth noting that on-line tracking is mandatory for some possible applications of baro-inertial technology, such as pre-impact fall detection, which may require on-line estimation of vertical velocity to design efficient detectors [23,26].

In conclusion, we have verified that Method B outperformed Method A during no-motion and forced circular motion tests. The height and vertical velocity RMSE values and the variance of the height RMSE values were lower when the whitening filter was used, compared with when it was not inserted in the conditioning block. Method B and Method A were nearly equivalent during free-fall and squat tests, although the use of the whitening filter might lead to precise, yet inaccurate height estimates for low-frequency motions.

We now compare the proposed sensor fusion methods with other methods reported in the literature on baro-IMUs that are customized for human motion tracking [5,8]. We are not aware of any research report using commercial baro-IMUs with proprietary sensor fusion software applied to human motion tracking.

Robust and accurate tracking was claimed in [5] using an integrated UWB baro-IMU sensor unit. In addition to the quaternion from the body frame to the navigation frame, height and vertical velocity of the tracked body and the bias of the inertial sensors, the sensor fusion method incorporated the barometric altimeter baseline in the state vector; this was done for better tracking the barometric altimeter measurement fluctuations as the atmospheric conditions change. In addition to the baro-IMU measurements, UWB-based range measurements were considered in either an EKF or a second-order EKF. One experimental scenario concerned normal height tracking in human motion: the motion to be tracked lasted about 85 s and consisted of vertical oscillations with peak-to-peak values of about two meters and frequencies of about 0.6 s. Given the presence of the UWB in the integrated solution, the results discussed in [5] cannot be directly compared to ours; however, it is instructive that the height RMSE went from 16 cm to 29 cm in the EKF solution during periods of UWB outage lasting ten seconds; the height RMSE went from 13 cm to 16 cm when the second-order EKF was tested using data from the same experiment as above.

In [8] two experiments focusing on application to indoors height tracking of human-like motions were designed to test and analyze the performance of a sensor fusion method employing a two-state Kalman filter that combined strap-down rotated vertical acceleration and barometric altimeter output. A stationary reference barometer was also deployed in the effort to improve the accuracy of height estimates. A market-available baro-IMU with proprietary EKF-based orientation algorithms was used for strap-down rotation; the two-state Kalman filter, to which the complementary filter implemented in this paper is identical when the gain **K***_c_* is chosen as in Equation (25) [19], was set with a process noise standard deviation *σ_w_* = 0.8 m*g* and a measurement noise standard deviation *σ_v_* = 1 m (process and measurement noises were modeled as mutually uncorrelated additive white Gaussian noises). The sensor fusion methods of this paper failed with this tuning to produce accurate estimates of height; the reason, we believe, was that the reliability of the linear acceleration *measurements* was overestimated, while underrating the reliability of the barometric altimeter measurements. In the first experiment described in [8], which consisted of an 8-s up-and-down motion of approximately ±0.5 m followed by a 10-s up-down motion of approximately ±1 m, the height error was bounded by ±0.2 m after correcting for the drift in barometric altimeter reading by differential barometry. The second experiment, in which the integrated sensor was strapped to the chest, showed “good accuracy and stability while walking up and down a staircase” for about one minute. Common to [5,8] is that no data were reported as for the accuracy of vertical velocity and one replication of each experiment was taken without statistical tests.

## 5. Conclusions

A loosely coupled filtering approach was developed in this paper for estimating the vertical velocity and height of a rigid body using baro-IMU sensor data. The sensor fusion method was based on a quaternion-based EKF for attitude estimation and strap-down rotation of the sensed specific force using inertial sensors, followed by a complementary filter fed with the estimated vertical linear acceleration and the output from the barometric altimeter. For validation purposes the sensor fusion method was implemented in MATLAB using our WIMU system, consisting of a baro-IMU paired via Bluetooth to a smart-phone, which was wirelessly interfaced to a PC running MATLAB via WiFi. Successful validation was achieved in different experimental conditions, which included no-motion, free-fall motion, forced circular motion and squatting. The porting of the sensor fusion method to the target WIMU controller is currently underway.

## Figures and Tables

**Table 1.** Parameter tuning.

**Process Noise Model Parameters**	
*σ_g_*, °/s	1
*^a^σ*, m/s^3^ (×10^−4^)	5
*α*, s^−1^	0
**Measurement Noise Standard Deviations**	

*σ_a_*, m*g*	10
*σ_w_*, m*g*	15
*σ_v_*, cm (Method A)	30
*σ_v_*, cm (Method B)	15
**Threshold of the Vector Selection Method**	

*λg*, m*g*	150

**Table 2.** RMSE statistics expressed as mean value ± standard deviation (SD) across *N* = 10 trials.

	**Height RMSE, m**	**Vertical Velocity RMSE, m/s**
Method A	0.40 ± 0.15 ***	0.07 ± 0.01 ***
Method B	0.08 ± 0.03 ***	0.02 ± 0.01 ***

**Table 3.** RMSE statistics expressed as mean value ± standard deviation (SD) across *N* = 10 trials.

	**Height RMSE, m**	**Vertical velocity RMSE, m/s**
Method A	0.07 ± 0.03	0.14 ± 0.05
Method B	0.05 ± 0.02	0.13 ± 0.04

**Table 4.** RMSE statistics expressed as mean value ± standard deviation (SD) across *N* = 10 trials.

	***f****_o_* **= 0.25 Hz**	***f****_o_* **= 0.5 Hz**	***f****_o_* **= 0.75 Hz**	***f****_o_* **= 1 Hz**	***f****_o_* **= 1.25 Hz**
**Method A**					
Height RMSE, m	0.38 ± 0.17 **	0.44 ± 0.24 **	0.54 ± 0.29 **	0.48 ± 0.22 ***	0.68 ± 0.19 ***
Vertical velocity RSME, m/s	0.08 ± 0.01 **	0.08 ± 0.01 **	0.11 ± 0.02 **	0.13 ± 0.02 ***	0.24 ± 0.06 ***
**Method B**					
Height RMSE, m	0.15 ± 0.02 **	0.10 ± 0.03 **	0.09 ± 0.03 **	0.10 ± 0.02 ***	0.68 ± 0.07 ***
Vertical velocity RMSE, m/s	0.06 ± 0.01 **	0.05 ± 0.01 **	0.08 ± 0.02 **	0.11 ± 0.02 ***	0.22 ± 0.06 ***
